# The Role of SPECT/CT and PET/CT Hybrid Imaging in the Management of Ectopic Thyroid Carcinoma—A Systematic Review

**DOI:** 10.3390/diagnostics14131369

**Published:** 2024-06-27

**Authors:** Claudiu Peștean, Alexandru Pavel, Doina Piciu

**Affiliations:** 1 Faculty of Medicine, “Iuliu Hațieganu” University of Medicine and Pharmacy, 400012 Cluj-Napoca, Romania; claupestean@yahoo.com (C.P.); doina.piciu@gmail.com (D.P.); 2 “Ion Chiricuță” Oncology Institute, 400015 Cluj-Napoca, Romania; 3 Affidea CT Clinic, 400015 Cluj-Napoca, Romania; 4 Emergency Clinical County Hospital Cluj-Napoca, 400347 Cluj-Napoca, Romania

**Keywords:** ectopic thyroid carcinoma, F-18 PET/CT, I-131 SPECT/CT, hybrid imaging

## Abstract

Background and Objectives: Thyroid ectopy represents a rare disease with an incidence of 0.3–1/100,000. It occurs due to the defective embryological process of the thyroid gland development. The thyroid ectopic tissue may suffer malignant transformation. This review aims to shed light on the roles that I-131 SPECT/CT (radioiodine 131 single-photon emission tomography fused with computed tomography) and F-18 PET/CT (fluorodeoxyglucose F18 positron emission tomography fused with computer tomography) may play in managing patients with ectopic thyroid carcinoma. Materials and Methods: A total number of 47 articles were identified on the PubMed and Google Scholar databases, and 3 other articles were selected from articles identified in the references cited in the retrieved articles. After refining the selection, the inclusion and exclusion criteria were applied, resulting in 10 articles that were included in the review. Results: The cases of ectopy included in this review were localised as follows: four cases in the thyroglossal duct, two cases in the mediastinum, one case in the oesophagus, one case in the thorax, one case with a pre-tracheal location, and one case with a latero-cervical location. In all the cases, F-18 FDG PET/CT was used as a diagnostic tool. In one case, F-18 FDG PET/CT was combined with I-131 SPECT/CT and MRI (magnetic resonance imaging). In one case, it was combined with 68 Ga-FAPI PET/CT (Ga-68 radiolabelled FAP inhibitor positron emission tomography fused with computer tomography). The maximum SUVs (standardised uptake values) ranged from 5.5 to 25 g/mL. Conclusions: F-18 PET/CT and I-131 SPECT/CT hybrid nuclear imaging is of great value in assessing ectopic thyroid carcinoma. F-18 FDG PET/CT plays an important role in the primary tumour evaluation and distant disease detection. Ga-68 FAPIs are a promising alternative. I-131 SPECT/CT adds important information related to the anatomical characterization of primary and distant iodine-avid lesions.

## 1. Introduction

The thyroid gland is the largest endocrine gland in the human body, playing an exclusive endocrine role and weighing up to 20–30 g, and human body balance depends on its normal functioning [1,2]. Normally located in the anterior part of the neck, the thyroid gland may undergo ectopic developments, with or without the coexistence of the orthotropic thyroid. Ectopic thyroid tissue has a prevalence from 7% to 10% according to autopsy studies [3]. Ectopic thyroid tissue is often associated with thyroid hypo- or hyperfunction that may lead to the development of goitre or thyroiditis, and that may be affected by benign or malignant transformation [3].

The thyroid carcinoma (TC) incidence has consistently increased in recent decades, with TC being the most common endocrine malignancy. It occupies the fifth place in the statistics covering diagnosed cancers among women [4,5], with a female-to-male ratio of approximately 3:1 [6]. 

This systematic review aims to shed light on the role that I-131 SPECT/CT (radioiodine 131 single-photon emission tomography fused with computed tomography) and F-18 FDG (fluorodeoxyglucose F18) PET/CT (positron emission tomography fused with computer tomography) may play in managing patients with ectopic thyroid carcinoma, according to the published data on this topic.

### 1.1. Embryology and Aetiology of Thyroid Ectopy

During the 24th day of gestation, the development of the thyroid begins as a proliferation process of the endodermal cells originating on the floor of the primitive pharynx, at the level of the foramen cecum, in a median position, between the first and second branchial arches. Between the fifth and seventh weeks of intrauterine life, the primitive thyroid migrates from its initial location while remaining connected to the foramen cecum through the thyroglossal duct, which is obliterated at the end of the migration. During this migration, the thyroid primordium passes through the tongue, penetrating the mesoderm, and it develops as a bilobular shape. This median primordium settles at the anterior neck in the seventh week of gestation. At this position, it connects with two lateral ultimobranchial bodies that emerged from endodermal cells initially located at the fourth pharyngeal pouch, and from ectodermal cells that were initially located at the fifth pharyngeal pouch. These lateral anlagen contain parafollicular C cells. This complex process explains two important aspects: the predominant distribution of parafollicular C cells in the lateral parts of the thyroid gland and the existence of lateral ectopic thyroid masses [3,7].

Ectopic thyroid is a rare condition, and this congenital malformation occurs in the general population with an incidence of 0.3–1/100,000 [8]. Less than 1% of all thyroid cancers occur in ectopic thyroid [9]. A total number of approximately 130 cases of ectopic thyroid carcinoma were documented and treated worldwide [8]. The most frequent histological types of ectopic thyroid carcinoma are papillary thyroid carcinoma and follicular thyroid carcinoma. The follicular variants of papillary thyroid carcinoma, medullary thyroid carcinoma, anaplastic thyroid carcinoma, and the insular subtype of papillary thyroid carcinoma are less frequent [9]. 

Migration defects are considered the most common causes of thyroid ectopy. Thyroid migration abnormalities may also be present when morphogenesis heart abnormalities occur, due to the proximity of the thyroid primordium and the heart. If the thyroglossal duct is not completely closed and degenerated, it may undergo cystic transformation or contain thyroid tissue [7]. 

Molecular and genetic mechanisms are also described in thyroid ectopy. Genetic studies have identified nineteen genes exclusively associated with thyroid ectopy. The most common mutations involved in thyroid ectopy are related to TITF-1 (thyroid transcription factor 1), Foxe1 (formerly called thyroid transcription factor 2), and PAX-8 (Paired-box gene 8) [3]. 

The presence of distant ectopic thyroid tissue may be explained by the aberrant migration of uncommitted endodermal cells [3].

Mediastinal or sub-diaphragmatic thyroid ectopy may be explained by an overdescended thyroglossal duct [3].

The most frequent sites of ectopic thyroid tissue are the lingual, sublingual, thyroglossal, laryngeal, tracheal, and latero-cervical sites. While extremely rare, ectopic thyroid tissue can be found remotely in the oesophagus, mediastinum, heart, pancreas, adrenal glands, gallbladder, and skin [9].

The most frequent type of ectopic thyroid carcinoma described in the literature is the papillary type. According to Fu et al., papillary thyroid carcinoma coexists with ectopic thyroid tissue in over 90% of the malignant transformations of thyroid ectopic cases. Klubo-Gwiezdzinska et al. reported the presence of papillary ectopic thyroid carcinoma in 80% of cases, and the follicular variant of papillary thyroid carcinoma in 8% of cases of thyroglossal duct ectopic thyroid carcinomas [9]. However, follicular, medullary, and anaplastic ectopic thyroid carcinoma cases have also been reported as coexistent in the literature [9].

### 1.2. SPECT/CT Hybrid Imaging in Thyroid Cancer

SPECT/CT hybrid imaging may be of great value in both eutopic and ectopic thyroid carcinoma cases due to a combination of two aspects. The first one is the existence of thyroid-related radiopharmaceuticals suitable for SPECT imaging, and the second is that hybrid imaging techniques have the advantage of dual perspectives, both functional and structural.

Radioiodine is the physiological element used for diagnostic and therapeutic assessments in thyroid pathologies. Iodine is highly metabolised in follicular cells, making it ideal for thyroid imaging. No matter what isotope is in discussion, the chemical behaviour of iodine in the organism follows the same pathway, and, in thyroid tissue, it exploits the same specific mechanism: thyroid hormone production. 

Radioiodine I-123 sodium iodide (I-123 NaI) is the preferred diagnostic radiopharmaceutical for thyroid imaging due to its pure gamma-emitting properties, with a physical half-life of 13.22 h and the most predominant photon energy at 159 and 127 keV. It has two major advantages: it is a pure gamma emitter and does not impose an unnecessary radiation burden caused by other emissions (e.g., beta particles), and it does not produce the stunning effect on thyroid tissue and does not affect future radioiodine ablation procedures in case of thyroid carcinoma [1,10]. Described initially by Rawson, the stunning effect is when the initial diagnostic dose of I-131 impairs and leads to the low uptake of the subsequent therapeutic dose of I-131 [11].

Radioiodine I-131 sodium iodide (I-131 NaI) is the most used radiopharmaceutical in the treatment of thyroid disease. Radioiodine therapy is essential for a differentiated thyroid carcinoma therapeutic approach, since it shows a clear benefit associated with an important decrease in mortality and a survival advantage for patients with advanced disease [12]. I-131 is a dual isotope, emitting beta particles suitable for therapy, 89.6% with an energy of 606 keV, and gamma photons used in diagnostic procedures, with an energy of 364 keV in 81.5% of cases and a half-life of 8.02 days [1,10].

If the above-mentioned radiopharmaceuticals are suitable for follicular-cell-derived thyroid carcinomas, in medullary thyroid carcinoma, a different uptake mechanism should be exploited similarly to pheochromocytomas and neuroblastomas, involving the ability of medullary thyroid carcinoma to produce catecholamines. Meta-iodo-benzyl-guanidine (MIBG) labelled with I-123 or I-131 can be used in hybrid imaging techniques for medullary thyroid carcinoma, as it is taken up and stored in the catecholamine vesicles of medullary thyroid carcinoma (MTC) cells [1,13].

In differentiated thyroid carcinoma, postoperative SPECT/CT diagnostic procedures may be considered, although there is a strong concern regarding the stunning effect of I-131, which can negatively impact radioiodine metabolic therapy. Therefore, a small amount of I-131 NaI (37–111 MBq) should be used, or I-123 NaI is preferable, with therapeutic sequencing performed within 72 h. This pre-therapeutic scan should be considered only when the surgical report, ultrasonographical result, and clinical examination are unable to provide sufficient data for a correct therapeutic decision about treatment and what activity to deliver. Diagnostic I-123 or I 131 SPECT/CT scans can be useful in the follow-up, with additional advantages over radioiodine whole-body scans (WBSs), providing a better anatomical characterization of the potential lesions than whole-body scans. Post-therapeutic I-131 SPECT/CT is of incremental value, since, due to its hybrid perspective, it offers a higher sensitivity of 78% and an unbeatable specificity of 100%. It has a good predictive value for persistence/recurrence risk. Post-therapeutic SPECT/CT has incremental value in those situations when uncertainties are present on post-therapeutic whole-body scans and the results are inconclusive [14].

In medullary thyroid carcinoma, despite the addressability of MIBG as a radiopharmaceutical for SPECT/CT, its use is limited due to the limited sensitivity of the method [15].

### 1.3. PET/CT Hybrid Imaging in Thyroid Cancer

PET/CT is a hybrid nuclear technique that has substantially changed the diagnostic strategies in many oncological pathologies since its introduction into clinical use. The radionuclides used in PET/CT are positron emitters, many with very short half-lives. Fortunately, in thyroid carcinoma, there are suitable PET radiopharmaceuticals with long-enough-physical-half-life radionuclides, making them available for transport over distance and facilitating their use. PET/CT in thyroid carcinoma primarily addresses questions about tumour behaviour beyond those addressed by the conventional radiopharmaceuticals used in SPECT/CT or planar scintigraphy. The most common radiopharmaceutical in PET/CT remains F-18 FDG, responsible for assessing the metabolic activity in tumour lesions. However, PET/CT in thyroid carcinoma may benefit from other radiopharmaceuticals, like I-124 NaI, F-18 NaF, G-68 DOTATE (DOTA-0-Tyr3-Octreotate), and F-18 DOPA (dihydroxyphenylalanine).

I-124 exploits the same pharmacokinetic pathway as other radioiodine compounds: thyroid hormone production. It has a half-life of 4.2 days, a characteristic that makes it suitable for imaging and dosimetric assessments [16]. Compared to I-131 SPECT/CT, I-124 PET/CT offers several advantages, including a better spatial resolution, better sensitivity, and no stunning effect [16]. 

F-18 remains the most used radioisotope in PET. It has a half-life of 110 min, and it is a pure positron emitter. F-18 FDG is taken up by malignant cells due to the overexpression of GLUT (glucose transporter) membrane transporters, and it is suitable for assessing abnormal metabolic activities. This feature makes the F-18 FDG an excellent radiopharmaceutical that can assess cancer detection, staging, and recurrence [17]. Most of the well-differentiated thyroid carcinomas are slow-growing, and F-18 FDG PET/CT may not detect the lesions. Thus, the role of this hybrid imaging method may not be indicated in the initial staging and tumour assessment but may play an important role in the follow-up. Apart from being an initial diagnostic tool for thyroid carcinoma, F-18 FDG PET/CT is a method that contributes incidentally to the early detection of malignant thyroid lesions and thyroid incidentalomas, which have had an increased prevalence since the wide spread of F-18 FDG PET/CT [18]. F-18 FDG PET/CT plays an important role in the follow-up of patients with elevated thyroglobulin levels and negative radioiodine whole-body scans. In cases of de-differentiation, the malignant thyroid cell changes its metabolic behaviour, and it reduces its capabilities to uptake radioiodine but increases its F-18 FDG uptake, the so-called “flip-flop phenomenon”. F-18 FDG PET/CT is a valuable method in the assessment of the differentiated thyroid carcinoma recurrence, especially in high-risk patients. Anaplastic thyroid carcinoma (ATC), a rapidly growing thyroid tumour, also benefits from F-18 FDG PET/CT in the initial staging in the evaluation of distant metastases, the evaluation of the treatment response to systemic or local therapy, and the follow-up. Sporadic MTC cases or MEN type 2B (multiple endocrine neoplasia type 2B) syndrome are better candidates than MEN type 2A (multiple endocrine neoplasia type 2A) syndrome for F-18 FDG PET/CT [14,16,19]. 

Other PET radiopharmaceuticals may be used for the diagnosis of thyroid carcinomas. F-18 DOPA PET/CT is the most specific molecular-imaging alternative for MTC [16]. 

Ga-68 DOTATATE PET/CT may detect MTC with somatostatin receptor expression and is a valuable hybrid diagnostic tool in this type of neoplasia in particular cases [16]. F-18 NaF PET/CT may be useful in detecting osseous and extraosseous metastases with calcifications of MTC [16].

Another novel and promising PET radiopharmaceutical may be used in head and neck cancers, including thyroid carcinoma targeting the cancer-associated fibroblasts, one of the most abundant cells in tumour stromata. Their presence in tumour stromata is associated with the high expression of the fibroblast-associated protein (FAP), a protein which is very limited in normal tissue. This makes PET imaging with Ga-68- or F-18-radiolabelled FAP inhibitors (FAPIs) a specific imaging tool in cancer disease; thus, Ga-68-labelled FAPIs have better pharmacokinetics and higher diagnostic accuracy [20,21]. The sensitivity of this novel tracer is superior to F-18 FDG in depicting neck lesions and distant metastases.

## 2. Materials and Methods

We performed a search on the PubMed and Goggle Scholar international databases using the following controlled keywords: “PET CT”; “SPECT CT”; “hybrid imaging”; “ectopic” and “thyroid carcinoma”; “lingual thyroid carcinoma”; “English publications”. The search was carried out using the controlled keywords included in five syntaxes, “SPECT CT hybrid imaging in ectopic thyroid carcinoma”; “PET CT in ectopic thyroid carcinoma”; “hybrid imaging in ectopic thyroid carcinoma”; “SPECT CT in lingual thyroid carcinoma”; and “PET CT in lingual thyroid carcinoma”, for a comprehensive search on the topic of the hybrid imaging and ectopic thyroid carcinoma association. The search process and the article selection/analysis were conducted during March 2024 and April 2024, aiming for articles from the earliest records to April 2024.

The inclusion criteria comprised articles discussing the role of hybrid nuclear imaging in ectopic thyroid carcinoma, full-text articles on the topic of interest, and English publications.

The exclusion criteria were articles not directly related to hybrid nuclear imaging and ectopic thyroid malignant disease, as well as preclinical and simulation studies.

Upon the retrieval of potentially eligible papers, the articles were analysed for their eligibility. A total of 47 articles were identified, and another 3 articles were identified by searching the reference list of the retrieved papers. We then identified and removed three duplicate articles, resulting in a total of 47 unique records for analysis. We further refined the selection, considering eligible only articles consisting of full-text original articles and literature reviews, articles involving human subjects, and articles adhering to the inclusion and exclusion criteria. We excluded 15 articles for reasons such as not being full-text articles, not being related to human subjects, or based on the title alone. Another 22 articles were excluded due to not meeting the exclusion criteria. 

**Figure 1:**
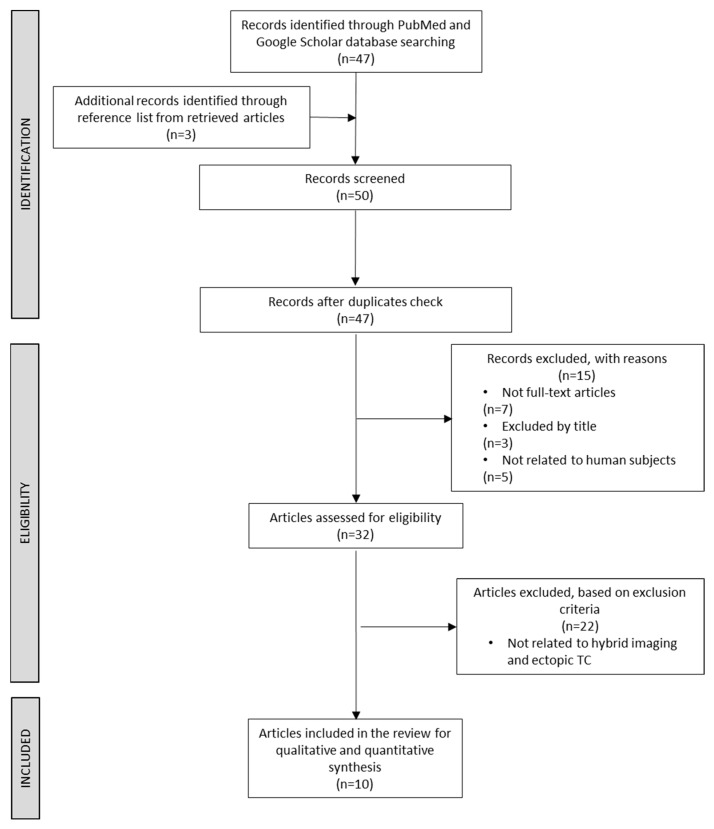
Selection criteria (PRISMA Flow Diagram).

Following the application of the selection and exclusion criteria, a total number of 10 articles were included in this systematic review, as presented in Figure 1.

We used Microsoft Excel 2021 for the data collection, table data organisation, and statistical analysis. 

## 3. Results

The articles selected as eligible for the present review included nine case reports and one case report with a literature review on the topic of ectopic thyroid carcinoma where hybrid nuclear imaging techniques were used as the diagnostic tools. A total number of 10 patients were summarised in the selected articles. The reports were published between 2011 and 2024.

The studies included in this review are listed in Table 1.

**Figure 2:**
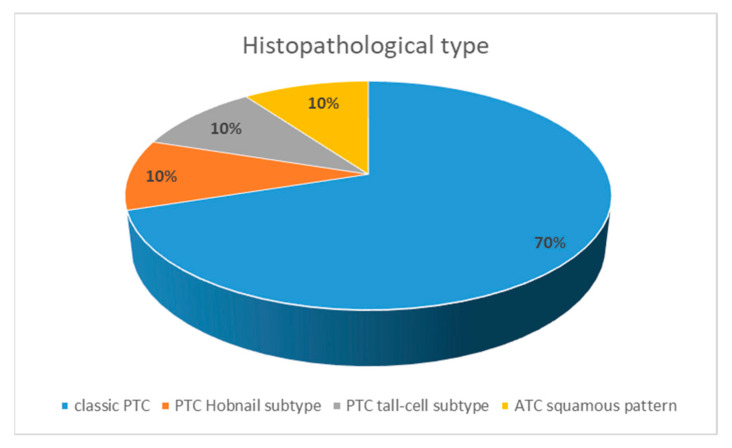
The histopathological types of the ectopic thyroid carcinoma cases.

Of these 10 articles, 7 articles describe cases of classic papillary thyroid carcinoma (70%), and the other 3 articles describe one case of papillary thyroid carcinoma (hobnail subtype) (10%), one case of papillary thyroid carcinoma (tall-cell subtype) (10%), and one case of anaplastic thyroid carcinoma with a squamous pattern (10%), as shown in Figure 2. 

**Figure 3:**
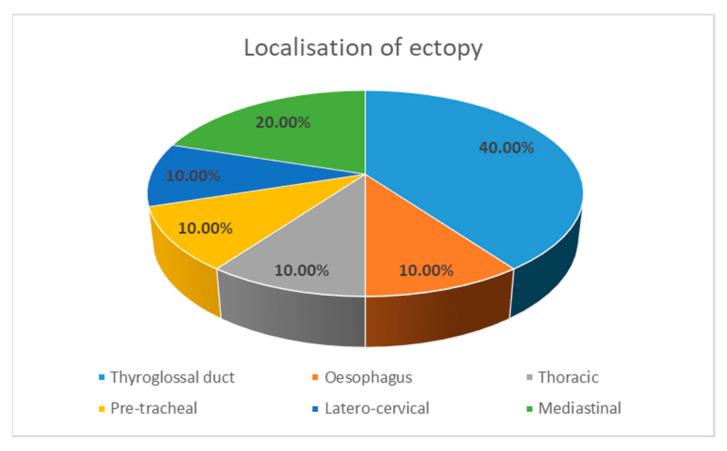
The localization of ectopy.

We underline that there were no follicular or medullary thyroid cancer cases in the ectopic presentation. The localization of the ectopic carcinomas revealed in these case reports was the thyroglossal duct in four cases (40%), the mediastinum in two cases (20%), the oesophagus in one case (10%), the thorax in one case (10%), a pre-tracheal location in one case (10%), and a latero-cervical location in one case (10%), as shown in Figure 3.

The median age of the patients included in the aforementioned case reports was 57 years, and the average was 54 years, ranging from 24 to 80 years. The female-to-male ratio was 7:3. 

All the patients underwent the resection of the ectopic tissue. A total of six patients underwent total thyroidectomy, and, in two cases, the presence of neoplastic disease in the orthotopic thyroid gland was mentioned. Five patients underwent selective lymphadenectomy; of these patients, one was free of lymph node metastatic involvement. 

A total of four cases had metastatic involvement as follows: two cases presented with latero-cervical lymph node metastases, one case had vertebral spine metastatic involvement, and another one had clavicular bone and lymph node metastases. Additionally, one case had synchronous pulmonary adenocarcinoma. 

Five patients were referred for I-131 radioiodine therapy. Of these patients, one refused the therapy, and four followed the procedure.

Adjuvant therapies were applied in two cases, like external-beam radiation therapy (EBRT) and systemic therapy with tyrosine kinase inhibitors (TKIs). 

These particular cases of ectopic thyroid carcinomas were investigated using different hybrid nuclear-imaging methods. In all cases, F-18 FDG PET/CT was used as a diagnostic tool to investigate the pathology involved (100%). In one case, F-18 FDG PET/CT was combined with I-131 SPECT/CT and F-18 PET coronal images were fused with MRI (magnetic resonance imaging) (10%). In another case, it was combined with 68 Ga-FAPI PET/CT (10%). 

Regarding F-18 FDG PET/CT investigation, not all the authors calculated and/or presented the SUVs (standardised uptake values) for detected lesions, but for those cases where they are mentioned, the maximum SUVs are high, ranging from 5.5 to 25 g/mL. 

F-18 FDG PET/CT was used in all 10 cases for the initial diagnosis. Other morphological imaging methods used for initial diagnosis included diagnostic computer tomography in six cases, ultrasonography in three cases, and MRI in three cases. Ga-68 FAPI PET/CT was also used for the initial diagnosis in one case.

We found details about the patient follow-ups only in a few reports. F-18 FDG PET/CT was mentioned in just one case as a follow-up diagnostic tool associated with diagnostic stand-alone CT and I-131 WBS. Neither I-123 nor I-131 SPECT/CT was mentioned in any case as a follow-up diagnostic tool. However, I-131 WBS was reported in two cases and I-123 in one case as follow-up imaging methods, without additional SPECT-CT. 

The data are summarised in Table 2. 

## 4. Discussion

We identified peculiar data concerning this pathology and the involvement of nuclear hybrid imaging in its diagnostic strategy. However, we found no large cohort studies or consistent research concluding the prognostic value of hybrid imaging in ectopic thyroid carcinoma. Nevertheless, Vázquez et al. mentioned in their case report that the volume of F-18 FDG-avid lesions and the SUV are good predictors of overall survival [24]. They also mentioned that the case they presented had a de-differentiation time of 6 months. F-18 FDG highly avid lesions in association with negative I-131 WBS were, in their opinion, a sign of de-differentiation. The time of de-differentiation is the second most important prognostic factor; patients with less than 3 years of de-differentiation time have a worse prognosis, as mentioned [24].

We observed in the present review that, among the ectopic thyroid carcinoma reports, the female-to-male ratio was 7:3. Other literature data mention an incidence of ectopic thyroid carcinoma in favour of women, with approximately 65—80% more cases than in men [8]. This ratio is lower than those in the case of orthotopic thyroid carcinomas, where women are up to three–four times more affected than men [14,32]. 

The median age of patients with ectopic thyroid carcinoma was 57 years. It is widely recognised that age is a risk factor for thyroid neoplastic pathology. Decision-makers consider the age of 55 years a pertinent threshold for disease risk assessment [14]. 

The most frequent location of ectopy that we identified was the thyroglossal duct. However, the literature mentions the lingual and sublingual thyroids as the most frequent sites of ectopy (up to 90% of cases) [3]. We hypothesise that, although rarely asymptomatic, due to the possible bleeding and deglutition impairments, the lingual thyroid is often diagnosed before the malignant transformation of ectopic tissue. This is not the case with the location of the thyroglossal duct, an anatomical site that may be overlooked by the patient.

Although thyroid carcinoma is usually a slow-growing neoplasm, with indolent behaviour in many cases, some aggressive histological types may require an aggressive treatment strategy [33]. The hobnail and tall-cell subtypes of PTC (papillary thyroid carcinoma) and anaplastic thyroid carcinoma with a squamous pattern were among the histological categories described in the case reports that we included in the present review considered as high risk. Therefore, special diagnostic and more aggressive therapeutic strategies should be applied in these cases [14,34].

An indicator of advanced disease is the presence of metastatic lesions. Among the 10 cases in discussion, three of them presented lymph node involvements. The presence of vertebral spine and clavicle metastases was described, according to Khthir et al. and Vázquez et al., respectively [24,31].

Adjuvant therapies like EBRT and TKI systemic therapy were used in two cases. This is in line with the therapeutic guidelines, corresponding to the tumour aggressiveness and disease severity, and the absence of curative surgical possibilities, due to critical-organ involvement or the vicinity [14,35].

F-18 FDG PET/CT, as an assessment tool for tumour aggressiveness, was the most frequent hybrid imaging diagnostic alternative. It has the primary purpose of clarifying the suspicion of cancer for the lesions in all cases. These lesions were previously clinically detected by morphological imaging techniques such as computer tomography, magnetic resonance imaging, or ultrasonography. F-18 FDG PET/CT is of significant value in differential diagnosis: it can assist with determining the extent of the malignant involvement, clarifying the presence of distant disease, and assessing the treatment response. 

Qi et al. reported the important value of F-18 PET/CT in the detection of ectopic thyroid carcinoma that was synchronous with a contralateral pulmonary nodule, the latter histologically confirmed as pulmonary adenocarcinoma. The adenocarcinoma was more F-18 FDG-avid than the ectopic thyroid carcinoma [29]. 

Radioiodine metabolic therapy was used in four cases, according to the histology, staging, and risk categories. When radioiodine is administrated, the post-therapeutic WBS can confirm the iodine avidity of the lesions solely, or with incremental specificity when I-131 SPECT/CT is associated [36]. 

Radioiodine therapy was applied in all four cases after total thyroidectomy; thus, there are data in the literature stating that even though surgery is traditionally used to remove ectopic thyroid tissue, radioiodine treatment can be considered a safe alternative to provide an effective treatment [37].

I-131 SPECT/CT is of significant value in the case of ectopic thyroid carcinoma because it can assess anatomically equivocal lesions and provide additional superior information compared to WBS. Even though it is not a routine examination in orthotopic thyroid carcinoma [14], I-131 SPECT/CT might be necessary more often in ectopic cases. We must highlight that if this diagnostic method is taken into consideration in the pre-therapeutic phase, the risk of stunning effects that might impair the radioiodine treatment should be kept in mind. Also, as some authors consider, the introduction of I-131 SPECT/CT added to post-therapy scans or to follow-up radioiodine scans will provide more accurate anatomic localization not only for malignant nodal or distant disease but also for benign thyroid remnant tissue of the physiological uptake [36]. 

Sachpekidis et al. reported the association of F-18 PET images with MRI images on coronal reconstruction used to better clarify the anatomical localization of the ectopic F-18 FDG-avid tissue. This provided a better anatomical characterization of the tumour located in the lateral thyroglossal duct cyst. 

Shi Y et al. reported the successful association of F-18 FDG PET/CT with Ga-68 FAPI PET/CT in the investigation of the case they reported. The ectopic lesion had a low F-18 FDG uptake but increased FAPI avidity. The histopathological report described high FAP expression in the tumour sample. This novel PET radiopharmaceutical is of great importance and is a promising tool in the evaluation of many cancers, including head and neck and thyroid cancers. It is based on the possibility of targeting tumoral FAP with FAP inhibitor-labelled positron emitters; Ga-68 seems to be the most promising so far. 

The limitations of this review are mainly due to very limited data pointing out the role of hybrid imaging in ectopic thyroid carcinoma. We observed a lack of information and unclear reports in the literature about the follow-up of this particular pathology and how hybrid imaging may contribute to the monitoring strategy. Another limitation of the present review is the absence of struma ovarii, a clinical presentation that may undergo malignant transformation. Malignant struma ovarii is a mature teratoma with a predominant histology of ectopic thyroid tissue with malignant findings. This is a gynaecological cancer with a particular approach, different symptomatology, and different monitoring strategy in comparison with the other ectopic thyroid carcinomas. 

## 5. Conclusions

Ectopic thyroid carcinoma is a rare disease, and peculiar data can be identified among the available published articles. 

The diagnostic and therapeutic approaches to this particular presentation of thyroid carcinoma may differ due to the lack of expertise and the variety of clinical presentations, which may influence decision-makers in cases of an unspecific symptomatology and objective findings.

Hybrid nuclear imaging is of great value in assessing equivocal findings. 

F-18 FDG PET/CT plays an important role in the primary tumour evaluation, distant disease detection, treatment response evaluation, and tumour aggressiveness quantification. 

Other PET radiopharmaceuticals may make useful contributions. Ga-68-labelled FAPI radiopharmaceuticals might play a determinant role in the diagnostic strategy of ectopic thyroid carcinoma due to their high sensitivity and increased accuracy, but further studies are needed to establish their role.

I-131 SPECT/CT adds important information related to the anatomical characterization of primary or distant iodine-avid lesions. 

I-131 may produce a stunning effect if it is used for diagnosis in the pre-therapeutic phase.

I-123 could be considered not only in initial iodine pre-ablative assessment but also during follow-up, since it does not produce stunning effects.

## Figures and Tables

**Table 1:** The articles included in the review.

NrCrt	First Author’s Name Location of Correspondence	Year of Publication	Hybrid Imaging
1	Wang C.P. [22] Taiwan	2011	F-18 FDG PET/CT
2	Sachpekidis C. [23] Switzerland	2017	F-18 PET and MRI fused images, F18 FDG PET/CT, I-131 SPECT/CT
3	Vázquez O.R. [24] USA	2018	F-18 FDG PET/CT
4	Moreno A.J. [25] USA	2019	F-18 FDG PET/CT
5	Kaida H [26] Japan	2019	F-18 FDG PET/CT
6	Gao R. [27] China	2019	F-18 FDG PET/CT
7	Lee A.K. [28] Philippines	2022	F-18 FDG PET/CT
8	Qi Y. [29] China	2022	F-18 FDG PET/CT
9	Shi Y. [30] China	2023	18 F-FDG PET/CT, 68 Ga-FAPI PET/CT
10	Khthir R.* [31] USA	2024	F-18 FDG PET/CT

* Case report with literature review.

**Table 2:** Characteristics of the reported cases.

Cases	Age Gender	Surgical Approach	Metastases	Orthotopic Thyroid Carcinoma	Stage	Histology	RAI Therapy	Adjuvant Therapy
Case 1	51 male	RET, TT, LA	NO	YES	I	Classic PTC	NO	NO
Case 2	69 male	RET, TT, LA	Latero-cervical lymph nodes	YES	II	PTC hobnail subtype	YES	NO
Case 3	68 female	RET, TT, LA, RM	Bone clavicle, latero-cervical lymph nodes	NO	IVB	Classic PTC	YES	EBRT, TKI
Case 4	63 male	RET, LA	NO	NO	IVA	ATC	NO	NO
Case 5	80 female	RET	NO	NO	I	Classic PTC	NO	NO
Case 6	43 female	RET	NO	NO	I	Classic PTC	NO	NO
Case 7	24 female	RET, TT	NO	NO	I	Classic PTC	Yes	NO
Case 8	50 female	RET, TT	NO *	NO	I	Classic PTC	NO **	NO
Case 9	28 female	RET, LA	Latero-cervical lymph nodes	NO	I	Classic PTC	NO	NO
Case 10	67 female	RET, TT	Vertebral spine	NO	IVB	PTC tall-cell subtype	YES	EBRT

TT—total thyroidectomy; LA—lymphadenectomy; RET—resection of ectopic tissue; RM—resection of metastasis; EBRT—external-beam radiation therapy; TKI—tyrosine kinase inhibitor; *—presence of synchronous pulmonary adenocarcinoma; **—the patient refused the I-131 radioiodine treatment.

## Data Availability

The authors confirm that the data supporting the findings of this study are available within the article.
